# Brain-Derived Neurotrophic Factor (Val66Met) and Serotonin Transporter (5-HTTLPR) Polymorphisms Modulate Plasticity in Inhibitory Control Performance Over Time but Independent of Inhibitory Control Training

**DOI:** 10.3389/fnhum.2016.00370

**Published:** 2016-07-29

**Authors:** Sören Enge, Monika Fleischhauer, Anne Gärtner, Andreas Reif, Klaus-Peter Lesch, Matthias Kliegel, Alexander Strobel

**Affiliations:** ^1^Department of Psychology, Technische Universität DresdenDresden, Germany; ^2^Department of Psychology, PFH Private Hochschule GöttingenGöttingen, Germany; ^3^Department of Psychiatry, Psychosomatic Medicine and Psychotherapy, University Hospital FrankfurtFrankfurt am Main, Germany; ^4^Division of Molecular Psychiatry, Laboratory of Translational Neuroscience, Department of Psychiatry, Psychosomatics, and Psychotherapy, University of WuerzburgWuerzburg, Germany; ^5^Department of Psychology, University of GenevaGeneva, Switzerland

**Keywords:** executive function training, response inhibition, neuronal plasticity, BDNF Val66Met, 5-HTTLPR

## Abstract

Several studies reported training-induced improvements in executive function tasks and also observed transfer to untrained tasks. However, the results are mixed and there is a large interindividual variability within and across studies. Given that training-related performance changes would require modification, growth or differentiation at the cellular and synaptic level in the brain, research on critical moderators of brain plasticity potentially explaining such changes is needed. In the present study, a pre-post-follow-up design (*N* = 122) and a 3-weeks training of two response inhibition tasks (Go/NoGo and Stop-Signal) was employed and genetic variation (Val66Met) in the brain-derived neurotrophic factor (BDNF) promoting differentiation and activity-dependent synaptic plasticity was examined. Because Serotonin (5-HT) signaling and the interplay of BDNF and 5-HT are known to critically mediate brain plasticity, genetic variation in the 5-HTT gene-linked polymorphic region (5-HTTLPR) was also addressed. The overall results show that the kind of training (i.e., adaptive vs. non-adaptive) did not evoke genotype-dependent differences. However, in the Go/NoGo task, better inhibition performance (lower commission errors) were observed for *BDNF* Val/Val genotype carriers compared to Met-allele ones supporting similar findings from other cognitive tasks. Additionally, a gene-gene interaction suggests a more impulsive response pattern (faster responses accompanied by higher commission error rates) in homozygous l-allele carriers relative to those with the s-allele of 5-HTTLPR. This, however, is true only in the presence of the Met-allele of *BDNF*, while the Val/Val genotype seems to compensate for such non-adaptive responding. Intriguingly, similar results were obtained for the Stop-Signal task. Here, differences emerged at post-testing, while no differences were observed at T1. In sum, although no genotype-dependent differences between the relevant training groups emerged suggesting no changes in the trained inhibition function, the observed genotype-dependent performance changes from pre- to post measurement may reflect rapid learning or memory effects linked to BDNF and 5-HTTLPR. In line with ample evidence on BDNF and BDNF-5-HT system interactions to induce (rapid) plasticity especially in hippocampal regions and in response to environmental demands, the findings may reflect genotype-dependent differences in the acquisition and consolidation of task-relevant information, thereby facilitating a more adaptive responding to task-specific requirements.

## Introduction

In recent times, studies on the effectiveness of training of executive functions, especially of working memory (WM) training have suggested substantial performance improvements from pre- to post-test sessions in the trained task and also training-related transfer to untrained WM tasks (Klingberg, 2010; Morrison and Chein, 2011). Moreover, training-related transfer to other executive functions such as task switching, inhibition, and interference resolution, and most notably to psychometric fluid intelligence has been reported (Persson and Reuter-Lorenz, 2008; Karbach and Kray, 2009; Chein and Morrison, 2010; Borella et al., 2013). These findings have fostered the assumption that targeted interventions could be applied to maintain or enhance executive function performance (Klingberg, 2010; Enriquez-Geppert et al., 2013). However, considerable methodological issues of cognitive training studies have been addressed that challenge the notion that training-related performance changes can readily be attributed to specific alterations in the trained function, and alternative explanations of training effects such as the influence of motivation or the role of practice and learning have been raised (Shipstead et al., 2010, 2012; Melby-Lervåg and Hulme, 2013; Redick et al., 2013).

However given the large interindividual variability in training effects within and across studies (see e.g., Zinke et al., 2014), an examination of potential moderators of the above-mentioned training effects seems warranted, especially with regard to moderators that bear the potential for mechanistic explanations for the development and stability of training and transfer effects in the brain. Specifically, this refers to the fact that training-related performance changes would necessarily require some sort of modification, growth, or differentiation at the cellular and synaptic level (i.e., neuronal or synaptic plasticity), notably in those neuronal networks and neurochemical systems that are deemed to be functionally relevant for executive functioning (Kelly and Garavan, 2005; Klingberg, 2010; Lövdén et al., 2010; Buschkuehl et al., 2012; Enriquez-Geppert et al., 2013).

In this regard, one of the key regulators that drive neuroplasticity is the brain-derived neurotrophic factor (BDNF), a brain-wide distributed neurotrophic protein that critically mediates the differentiation, proliferation, and survival of subcortical and cortical neurons (Poo, 2001; Binder and Scharfman, 2004; Lessmann et al., 2004; Cohen-Cory et al., 2010). BDNF has been implicated in the growth of neurons, in axonal and dendritic morphogenesis, and in activity-dependent changes of synaptic plasticity, notably in the hippocampal formation and in cortical areas. Indeed, there is firm evidence that BDNF regulates long-term potentiation (LTP; Lessmann et al., 2004; Pang and Lu, 2004). Consequently, BDNF signaling in fronto-hippocampal networks has an established role in learning and memory acquisition, formation, and consolidation, as evidenced by a plethora of studies in animals and humans (Alonso et al., 2002; Monteggia et al., 2004; Bekinschtein et al., 2008, 2014; Cunha et al., 2010; Hoefer et al., 2014). Furthermore, BDNF impacts on the development and integrity of preforntal cortex (PFC)-dependent executive functions (Dincheva et al., 2012; Oral et al., 2012; Sakata et al., 2013) not only in the developing, but also in the adult brain, such as by promoting rapid synaptic plasticity as a function of experience and environmental demands (Poo, 2001; Cohen-Cory et al., 2010).

In addition, considerable support for the role of BDNF-induced plasticity in higher-order cognition and executive functioning comes from molecular genetic studies on the role of *BDNF* Val66Met, a functional single-nucleotide polymorphism (SNP, rs6265) in the *BDNF* gene (Egan et al., 2003; Bath and Lee, 2006; Dincheva et al., 2012). This polymorphism constitutes a conversion of valine (Val) into methionine (Met) in the pro-peptide of BDNF, which alters dendritic trafficking, synaptic localization, and BDNF secretion, with the Met-variant being functionally associated with decreased activity-dependent BDNF secretion (Egan et al., 2003; Chen et al., 2004). Consequently, the presence of one or two Met-alleles has frequently been associated with deficits in cognitive performance, notably in learning and memory tasks (Egan et al., 2003; Hariri et al., 2003; Goldberg et al., 2008) or tasks depending on the integrity of WM, top-down selective attention, and interference control (Schofield et al., 2009; Dincheva et al., 2012). Concomitant with these behavioral findings, Met-allele carriers exhibit structural and functional alterations in hippocampal and prefrontal areas relative to those homozygous for the Val-allele, such as smaller gray matter volume (e.g., Pezawas et al., 2004; Szeszko et al., 2005) and aberrant neural activity patterns in these regions (see Bath and Lee, 2006; Dincheva et al., 2012).

Given that BDNF promotes neuronal differentiation and rapid, activity-dependent synaptic plasticity in adult brain regions critically involved in higher-order cognition and executive control, BDNF appears to be an important moderator of training-related performance changes. Thus, in the present study, the role of BDNF was investigated in a sample of 122 healthy individuals that were administered to an inhibitory control (IC) training regime. Data on this sample were already reported in a previous article (Enge et al., 2014a). In this previous study, the effectiveness of IC training was examined by using Go/NoGo and Stop-Signal tasks that primarily tap into the response inhibition aspect of IC. Dramatic increases in task performance from pre- to post-testing sessions were observed that even persisted for months, but, in line with a number of recent studies (Shipstead et al., 2012; Melby-Lervåg and Hulme, 2013; Redick et al., 2013), no significant differences between the relevant training groups (i.e., the adaptive training group vs. active controls) were detected, but only a significant difference between the adaptive training group and the passive control group occurred. However, we did not examine whether training effects attributable to changes in the IC function (i.e., as a result of training-induced plasticity) might have been detected if important moderators of brain plasticity such as genetic variation in the *BDNF* gene (i.e., *BDNF* Val66Met) had been considered.

Therefore, these previous data were reanalyzed by considering gene variations as potential moderators. Specifically, we assumed that: (a) *BDNF* Val66Met genotypes would explain task-related performance changes; and (b) that *BDNF* genotypes would drive training-related changes differentially between the experimental groups, depending on the extent of training and genotype. In general, the Val/Val genotype was expected to result in superior task performance than compared to the Met-allele of *BDNF* Val66Met.

Furthermore, because serotonergic (5-HT) signaling is critically involved in neuroplastic changes in cortical and hippocampal brain regions during development and adulthood (Martinowich and Lu, 2008; Homberg et al., 2014) and plays an essential role in impulse control (Lesch and Merschdorf, 2000; Cools et al., 2008), training-related differences in the IC tasks were further expected to depend on variability in 5-HT function. To address this issue, genetic variation in the gene coding for the 5-HT transporter (5-HTT) was considered, which is a key regulator of 5-HT system function by mediating the active reuptake of 5-HT. The gene coding for the 5-HTT contains a prominent variation in its promoter region that leads to functional alterations of transporter function—the so-called 5-HTT gene-linked polymorphic region (5-HTTLPR). The short (s)-allelic variant of this polymorphism has been linked to lower transcriptional efficiency and lower 5-HTT function relative to the long (l)-allele (Lesch et al., 1996; David et al., 2005). Empirical data and recent theorizing suggests that the s-allele is associated with a higher sensitivity for and enhanced processing of motivationally relevant signals, such as contextually salient or threat-provoking stimuli as well as goal- or task-relevant information. This higher sensitivity, concomitant with increased attention or vigilance in s-allele carriers may partly result from a hyperreactivity of frontal cortical and subcortical regions such as the anterior cingulate, the medial prefrontal cortex (mPFC) and the amygdala. In terms of negative emotional information, this may lead to a higher vulnerability for mood disorders, notably under adverse environmental conditions. In the context of cognition, however, an increased processing of motivationally relevant stimuli could lead to benefits in cognitive performance (for an overview, see Homberg and Lesch, 2011). Indeed, an increasing number of studies in humans and animals using a range of executive function tasks suggest enhanced attention to goal-relevant information and beneficial performance in s-allele carriers relative to those homozygous for the l-allele (see e.g., Homberg and Lesch, 2011; Enge et al., 2014b; Gloster et al., 2015). Based on such findings, one would assume that s-allele carriers would exhibit superior task performance in the trained inhibition paradigms. Moreover, ample evidence suggests that 5-HT signaling shapes neuroplasticity in both the developing and the adult brain and that the interplay of BDNF and 5-HT signaling impacts on neuronal function and synaptic plasticity depending on experience and environmental demands (Murphy et al., 2003; Mattson et al., 2004; Ren-Patterson et al., 2005; Martinowich and Lu, 2008; Homberg et al., 2014).

Taken together, the present study sought to investigate the moderating role of genetic variation in training-induced performance changes. Specifically, we expected performance improvements particularly for individuals being homozygous for the Val-allele of BDNF and in carriers of the s-allele of 5-HTTLPR.

## Materials and Methods

### Participants and Procedure

Prior to the beginning of the study, all participants received written and oral information about the procedure and the aims of the study and all participants gave written informed consent prior to the beginning of the study that could be withdrawn anytime without giving any reasons, which conforms to ethical standards as well as the recommendations of the Department of Psychology of the Technische Universität Dresden. All data were collected and processed anonymously (pseudonymization). To match data, participants generated a code that only themselves can attribute to their person. Participants were fully debriefed after completion of the study and thanked for their participation.The sample comprised of 122 student volunteers (17 men, age: *mean* ± *SD* 21.3 ± 4.16 years, range: 18–38 years) who received course credit for their participation. All participants were of middle European ancestry and reported German as their mother tongue. Further, all participants had normal or corrected-to-normal vision and reported no relevant current health problems as well as no history of neurologic or psychiatric diseases, psychopharmacological treatment, substance abuse or dependence. The participants conducted a pre-test session (T1), a post-test session (T2) 4 weeks later, and a follow-up session (T3) 4 months after T2. In between T1 and T2, two of the experimental groups conducted nine training sessions (for a detailed description, see below). The pre-, post-, and follow-up test session each lasted about 2 h. Individuals completed a measure of fluid intelligence and several questionnaires such as assessing sociodemographics, mood, sleep duration and health status. After a short break, individuals then conducted three tasks measuring IC (Go/NoGo, Stop-Signal, and Stroop) that were presented in counterbalanced order. At the end of the pre-test session, saliva samples were collected for DNA extraction.

To investigate whether the training of the IC tasks leads to performance changes in the post- and follow-up sessions, at the end of the pre-test session, participants were randomly assigned to either one of two experimental groups that trained Go/NoGo and Stop-Signal tasks within a 3-week training period with nine training sessions between pre- and post-test session: (a) an adaptive training group (*n* = 43), in which task difficulty of the two training tasks was individually adjusted on a trial-by-trial basis; (b) a non-adaptive group (*n* = 39) in which task difficulty was fixed and related to the individual’s performance level obtained at the pre-test session. Because members of the non-adaptive group performed far below their capacity limit during the study, these individuals served as active controls. Moreover; and (c) a passive control group (*n* = 40) was formed by random assignment that did not receive training between pre- and post-test session, to provide comparison with other studies (see Enge et al., 2014a).

Note that there was a dropout of two individuals in the post-test session and of 11 individuals in the follow-up session 4 months after T2. Chi-square difference test revealed that drop-out was not significantly related to training or control group membership (*p* < 0.10). Genotyping was not successful for five individuals. Given the fact that training-related effects were very similar for T2 and T3 (see Supplementary Material, see also Enge et al., 2014a), we used a mean value for T2 and T3 task performance within the statistical analyses to increase statistical power resulting in 115 individuals (17 men, age *mean* ± *SD* 21.3 ± 4.15 years, range 18–38 years) being considered for subsequent analyses.

### Measures

In the following, those measures are described that are relevant for the present study aiming to investigate the moderating role of genetic variation in plasticity genes on training-related performance changes in the trained tasks. For a detailed description of measures, please see Enge et al. (2014a).

#### Training Tasks

A Go/NoGo and a Stop-Signal task were administered using the Presentation software^1^ running at LCD screens with a resolution of 1024 × 768 and were conducted for about 10 min each.

##### Go/NoGo Task

In the Go/NoGo task used in the pre-, post- and follow-up session, a sequence of black capital letters (A to Z) was presented in the middle of a white screen. Each stimulus was preceded by a fixation cross and occurred for 750–1250 ms. Participants were instructed to respond to any letter except “X” by pressing the “↓”-button as fast as possible (Go trial), but not to respond when the letter “X” occurred (NoGo trial), which was the case in 20% of trials. For the adaptive training and non-adaptive (active control) group, in the first training session, stimulus durations and NoGo stimulus rates were adjusted based on the individual’s pre-testing performance (stimulus duration: mean reaction time (RT) + 250 ms; NoGo stimulus rate in %: 20 ± factor reflecting the relation of correct rejections and false alarms; algorithm available upon request). Extreme values were adjusted to a stimulus duration of at least 350 ms and at maximum 1000 ms as well as a NoGo stimulus rate of at least 5% and not more than 35%. For the non-adaptive (active control) group, these parameters were used as fixed values for all training sessions. In the adaptive training group, however, where individuals trained the task corresponding to their performance development, the starting values at the beginning of the first training session were subsequently adjusted on a trial-by-trial basis. Stimulus duration decreased (increased) by 50 ms, if the participant’s response was correct (incorrect) or if the participant’s response was faster (slower) than the average reaction in the session’s previous trials. A lower threshold of 250 ms stimulus duration was determined to guarantee stimulus detection. Additionally, the NoGo stimulus rate decreased (increased) by 5%, if a NoGo stimulus was correctly ignored (incorrectly responded to), limited by a lower and higher threshold of 2.5 and 50%, respectively. Performance was saved at the end of each training session to provide a starting value for the next session. Note that the 2.5–50% NoGo rate variation and the 250–1000 ms range of stimulus presentation only refer to the adaptive training sessions and that the values represent predefined lower and upper limits of these task parameters. At the pre-, post- and follow-up test sessions (T1, T2/T3), these parameters were not adapted according to the participants’ individual performance but were the same for all participants to enable examining potential performance differences (after the training) between all experimental groups. This is also true for the Stop-Signal task outlined below.

##### Stop-Signal Task

In the Stop-Signal task used in the pre-, post-, and follow-up sessions, a series of black capital letters was presented in the middle of a white screen. Each letter was preceded by a fixation cross and occurred for up to 1000 ms. Participants were instructed to discriminate between vowels and consonants (presented in equal number) by pressing the buttons “←” (for vowel) or “→” (for consonant). In addition, participants were instructed to suppress their response if the letter appeared in red font color or changed its color from black to red (stop-signal) during the trial (stop-trial), which was the case in 25% of trials. In stop-trials the stop-signal delay (SSD) varied from 0 to 500 ms in 100 ms increments. In the first training session, SSD was adjusted according to the individuals’ pre-testing performance (based on mean RT and the relation between correct rejections and false alarms; algorithm available upon request). For participants of the non-adaptive group, this value was used during all training sessions. In contrast, for the adaptive training group, the value was used at the beginning of the first training session and was subsequently adjusted on a trial-by-trial basis by increasing (decreasing) SSD by 50 ms, if participants correctly (erroneously) executed the discrimination-task during a stop-trial. Performance indicators were saved at the end of each training session to serve as starting parameters in the following session.

#### Potential Confounding Factors

##### Fluid Intelligence

For the present study, the Raven’s Advanced Progressive Matrices (APM, Raven, 1990a,b) was used as a measure of fluid intelligence to control for possible differences between the genotype groups in fluid intelligence at pre-testing.

##### Mood States

Because individual differences in positive and negative affective states can impact on task engagement and performance, the participant’s current mood states were rated on the German version of the Positive and Negative Affect Schedule State version (PANAS-S, Krohne et al., 1996) at the beginning of each session.

##### Further Factors

Additionally, sex, age, malaise, sleep duration, and smoking status as well as caffeine and alcohol consumption during the past 24 h were assessed via questionnaire.

#### Genotyping

DNA was extracted using the Oragene^TM^ DNA Self-Collection Kit (DNA Genotek Inc., Ottawa, ON, Canada). *BDNF* genotypes were determined by routine polymerase chain reaction (PCR) followed by digestion of the PCR products and agarose gel size fractionation as described in detail elsewhere (Hunnerkopf et al., 2007; Mühlberger et al., 2014). The sample comprised 38 participants carrying at least one BDNF 66Met allele (35 Val/Met and 3 Met/Met carriers) and 77 participants homozygous for the Val66 allele (Val/Val genotype carriers). In accordance with previous studies, subjects with one or two copies of the Met allele were grouped together and contrasted to homozygous Val allele carriers (for review see e.g., Hariri et al., 2003; Goldberg et al., 2008; Dincheva et al., 2012).

5-HTTLPR was genotyped by PCR amplification according to a previously published protocol (Lesch et al., 1996). Additionally, using the protocol described by Wendland et al. (2006), a functional SNP within the 5-HTTLPR l-allele was determined with an A to G substitution (rs25531) of minor allele frequency (>9%), designated LA and LG (Nakamura et al., 2000). Homozygous s-allele, LG/LG and s/LG-cases were collapsed (e.g., Hu et al., 2006), and reclassified as s/s genotype (*n* = 28; 25%). Both heterozygous s/LA-cases and LA/LG-cases were reclassified as s/l genotype (*n* = 54; 50%). Carriers with two copies of the LA-allele are referred to as l/l genotype (*n* = 33; 25%). In accordance with previous studies (e.g., Lesch et al., 1996; David et al., 2005), subjects with one or two copies of the s-allele (s+, *n* = 82) were grouped together and contrasted to l/l genotype carriers.

Genotypes of 5-HTTLPR and BDNF Val66Met were in Hardy–Weinberg equilibrium and did not differ between the three experimental groups (all *p* > 0.10). Note that a small number of other polymorphisms were genotyped, which, however, were not relevant for the present study, but for other research questions such as the relationship of genetic differences to personality traits and mood.

### Statistical Analyses

To examine whether *BDNF* Val66Met and 5-HTTLPR modulate performance changes in the IC tasks in relation to the group membership individuals were assigned to, mixed-model analysis of variance (ANOVAs) were conducted using SPSS Statistics 21 (IBM Germany, Ehningen, Germany). In accordance to Enge et al. (2014a), performance changes were investigated for the Go/Nogo and Stop-Signal task separately using mean RT on Go-trials as well as more specific indicators of response inhibition (commission error rate in NoGo trials)^2^ at T1 and T2/T3 as within-subject factors. *BDNF* Val66Met and 5-HTTLPR as well as the group factor (i.e., adaptive, non-adaptive, and passive control group) were entered as between-subject factors. In the case of training-related effects in relation to the genetic variations, we would expect three way interactions (time × group × genotype) with genotype related differences in the training groups (but not in the passive control) at T2/T3 (but not at T1). Note that collapsing T2 and T3 task performance was motivated by the aim to maximize sample size and to reduce the number of single comparisons. It was justified by the fact that training-related effects were very similar for T2 and T3 (see Supplementary Material, see also Enge et al., 2014a).

As four different ANOVA models were conducted (Go/Nogo: RT, commision error; Stop-Signal: RT, commision error), correction for the multiple ANOVAs is necessary. Because the four depended variables were intercorrelated (|*r*| 0.32–0.92 at T1 and |*r|* > 0.22–0.90 at T2/3), a Bonferroni correction would have been too conservative to account for multiple testing, as Bonferroni correction requires independent variables. Therefore, the equivalent numbers of independent variables at T1 and T2/T3 were determined using the matrix spectral decomposition approach (Nyholt, 2004; Li and Ji, 2005). The mean equivalent number of independent variables was 3.15, therefore the significance threshold was adjusted to α = 0.05/3.15 = 0.016. In the “Results” Section, only main and interaction effects that meet this significance level will be reported.

Greenhouse-Geisser corrected degrees of freedom were applied where appropriate and original degrees of freedom, epsilon adjustment values, and corresponding *F* values were reported. Note that individuals with extreme values (i.e., values more than three times away from the interquartile range) in RT or commission error rates were discarded from the analyses (*n* = 4 in the Go/Nogo task).

## Results

### Between-Group Differences of Possible Confounding Factors

As was shown by Enge et al. (2014a), there were no systematic differences between the training groups (adaptive, non-adaptive, passive control group) in sex, age, fluid intelligence, as well as positive and negative affective mood states. Moreover, no systematic differences were observed with regard to sleep duration, smoking status, malaise as well as caffeine and alcohol consumption (all *p*s > 0.10). In the present study, we additionally tested for possible differences between the genotype groups in the potentially confounding factors. For 5-HTTLPR s+ allele carriers, we observed a trend towards a higher sleep duration at T1 (*p* = 0.078) and T3 (*p* = 0.052). However, as these effects were only marginally significant and as there were no associations between sleep duration and the dependent variables (all *p*s > 0.10), we did not include this factor as covariate. In all other tested factors neither 5-HTTLPR s+ allele carriers differed from the l/l genotype, nor did the *BDNF* Val/Val genotype group differed from *BDNF* Met-allele carriers (all *p*s > 0.10).

### Go/NoGo Task

**Figure 1:**
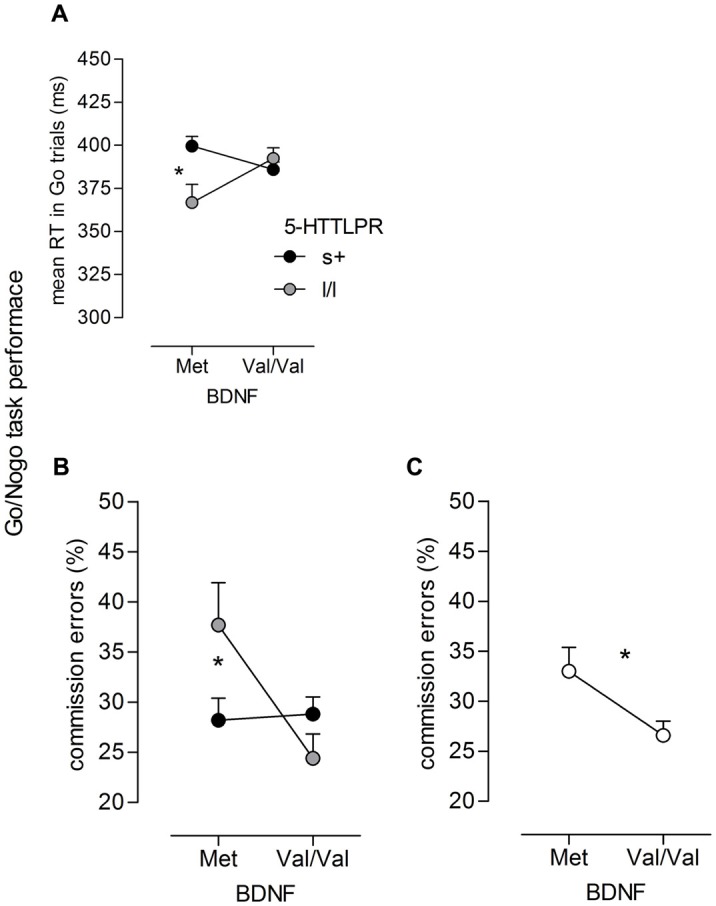
**Go/NoGo task performance.** Significant interactions of **(A)** 5-HTT gene-linked polymorphic region (5-HTTLPR) × brain-derived neurotrophic factor (BDNF; *F*_(1,99)_ = 7.76, *p* = 0.006, $\eta_{\text{P}}^{2}$ = 0.07) on mean reaction time (RT) in Go trials as well as **(B)** interaction of 5-HTTLPR × BDNF (*F*_(1,99)_ = 6.34, *p* = 0.013, $\eta_{\text{P}}^{2}$= 0.06) and **(C)** main effect of BDNF (*F*_(1,99)_ = 5.29, *p* = 0.024, $\eta_{\text{P}}^{2}$= 0.05) on commission error rate (in %), the effects occurred independent of the time of measurement; *n*_s+/Met_ = 28, *n*_ll/Met_ = 8, *n*_s+/ValVal_ = 51, *n*_ll/ValVal_ = 24; *p < 0.05, **p < 0.01.

For mean RT on Go trials, a significant time main effect (*F*_(1,99)_ = 239.55, *p* < 0.001, $\eta_{\text{P}}^{2}$ = 0.71) and a time × group interaction were revealed (*F*_(1,99)_ = 7.55, *p* < 0.001, $\eta_{\text{P}}^{2}$ = 0.13). As reported in more detail in Enge et al. (2014a), these effects indicate a large RT decrease from T1 to T2/T3 and a steeper slope for the training groups (adaptive training group, active control) than for the passive control group. With respect to geneotype-related performance difference, the mixed model revealed a 5-HTTLPR × *BDNF* interaction (*F*_(1,99)_ = 7.76, *p* = 0.006, $\eta_{\text{P}}^{2}$ = 0.07). There was no difference between 5-HTTLPR s+ allele and l/l genotype carriers carrying the *BDNF* Val/Val genotype (*p* > 0.10). In contrast, for *BDNF* Met-allele carriers, a significant difference between 5-HTTLPR genotypes occurred (*p* = 0.008), suggesting lower RTs for homozygous l-allele carriers compared to s-allele carriers of 5-HTTLPR (see Figure 1A).

Comission error rate (in %) was higher at T2/T3 than at T1 as indicated by a significant main effect of Time (*F*_(1,99)_ = 30.12, *p* < 0.001, $\eta_{\text{P}}^{2}$ = 0.23). With respect to genotype-related effects, again, a significant 5-HTTLPR × *BDNF* interaction was observed (*F*_(1,99)_ = 6.34, *p* = 0.013, $\eta_{\text{P}}^{2}$ = 0.06). As depicted in Figure 1B, carriers of the 5-HTTLPR s-allele showed lower errors of commission than l/l individuals when they carried the *BDNF* Met-allele (*p* = 0.047), while no significant difference between l/l and s+ individuals occurred when they carried the *BDNF* Val/Val genotype (*p* > 0.10). Together with the analysis on RT, the results show that the RT benefit of homozygous l-allele carriers of 5-HTTLPR occurred at the cost of more errors of commission or false alarms, respectively. Moreover, the analysis revealed a main effect of *BDNF* (*F*_(1,99)_ = 5.29, *p* = 0.024, $\eta_{\text{P}}^{2}$ = 0.05) indicating a significantly lower false alarm rate on NoGo stimuli for individuals carrying the Val/Val genotype than for those with the Met-allele (see Figure 1C).

Given our assumption of a possible moderating role of genotypes in training-induced improvements in executive functioning, we expected Time (T1, T2/T3) × group (adaptive, nonadaptive, and passive group) × genotype effects to occur in the ANOVAs. However, with respect to the Go/Nogo task, no such interaction occurred (all *p*s > 0.10).

### Stop-Signal Task

**Figure 2:**
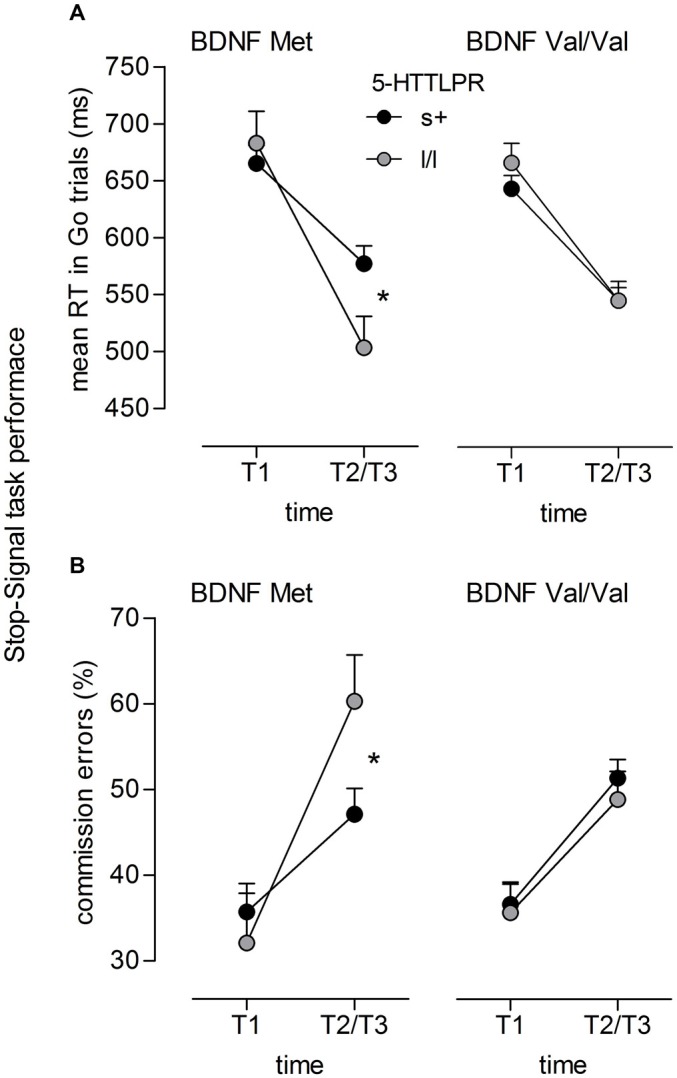
**Stop Signal task performance.** Significant interaction of **(A)** Time × 5-HTTLPR × BDNF (*F*_(1,103)_ = 6.49, *p* = 0.012, $\eta_{\text{P}}^{2}$ = 0.06) on mean RT in Go trials; and of **(B)** Time × 5-HTTLPR × BDNF (*F*_(1,103)_ = 9.48, *p* = 0.003, $\eta_{\text{P}}^{2}$ = 0.08) on commission error rate (in %); *n*_s+/Met_ = 29, *n*_ll/Met_ = 9, *n*_s+/ValVal_ = 53, *n*_ll/ValVal_ = 24; *p < 0.05.

For mean RT on Go trials, again a significant time main effect occurred (*F*_(1,103)_ = 323.56, *p* < 0.001, $\eta_{\text{P}}^{2}$ = 0.76) indicating lower RT in the post-test than in the pre-test session. As already reported by Enge et al. (2014a), this decrease in RT was larger for the training groups than for the control group as indicated by a significant time × group interaction (*F*_(2,103)_ = 8.59, *p* < 0.001, $\eta_{\text{P}}^{2}$ = 0.14). With respect to genotype groups, a highly significant Time × 5-HTTLPR interaction was observed (*F*_(1,103)_ = 17.82, *p* < 0.001, $\eta_{\text{P}}^{2}$ = 0.15). This effect was further qualified by a significant Time × 5-HTTLPR × *BDNF* interaction (*F*_(1,103)_ = 6.49, *p* = 0.012, $\eta_{\text{P}}^{2}$ = 0.06). While no genotype-related differences occurred at T1 (all *p*s > 0.10), lower RTs were observed for homozygous l-allele carriers of 5-HTTLPR relative to s-allele carriers at T2/T3. This difference between 5-HTTLPR genotype groups, however, only emerged for individuals that also carried the *BDNF* Met-allele (*p* = 0.021), while parallel to the Go/NoGo results, no difference between s+ and l/l genotypes was observed for Val/Val carriers of *BDNF* (*p* = 0.99; see Figure 2A).

Similar interactions occurred for commission error rate. Beside a main effect of time (*F*_(1,103)_ = 128.53, *p* < 0.001, $\eta_{\text{P}}^{2}$ = 0.56) indicating an increase in commission errors from T1 to T2/T3, again, a significant Time × 5-HTTLPR interaction occurred (*F*_(1,103)_ = 6.71, *p* = 0.011, $\eta_{\text{P}}^{2}$ = 0.06). This interaction was qualified by a significant Time × 5-HTTLPR × *BDNF* interaction (*F*_(1,103)_ = 9.48, *p* = 0.003, $\eta_{\text{P}}^{2}$ = 0.08) indicating no genoytpe-related differences at T1 (all *p*s > 0.10) but at T2/T3. Here, however, the interaction suggested better T2/T3 performance (i.e., lower false alarms in response to Stop trials) for individuals carrying the s-allele compared to those with the l/l genotype. This significant differences between 5-HTTLPR genotype groups, again, only occured for individuals carrying the *BDNF* Met-allele (*p* = 0.034), while no significant difference (*p* = 0.525) was revealed for individuals with the *BDNF* Val/Val genotype (see Figure 2B). In both ANOVAs adressing Stop-Signal task performance, no group-related interactions emerged (all *p* > 0.10)^3^.

## Discussion

Although training-induced changes in brain plasticity are widely assumed as primary explanatory mechanisms of possible training and transfer effects, neurotrophic factors known to directly promote neuronal plasticity in response to environmental demands have not yet been systematically examined in the context of executive function training. To this end, the main goal of the present study was to examine whether performance changes in two widely used IC paradigms might be explained by training-induced plasticity, as moderated by functional genetic differences in: (a) BDNF signaling (*BDNF* Val66Met); and (b) 5-HT function (5-HTTLPR).

The results provide evidence for a general role of genetic variation in BDNF function in explaining performance differences in the IC tasks. In view of the Go/NoGo task, homozygous carriers of the Val-allele showed better task performance than those carrying the Met-allele, as expected. This is suggested by a significant *BDNF* main effect that points to lower commission errors (false alarms) for Val/Val genotype carriers compared to Met-allele ones (which did not occur at the cost of RT). The role of *BDNF* in Go/NoGo performance was further demonstrated by interactions between *BDNF* Val66Met and 5-HTTLPR. For individuals carrying the Met-allele of *BDNF*, homozygous l-allele carriers of 5-HTTLPR showed shorter response latencies than s-allele carriers. However, in view of a similar gene interaction for error rate, this apparent response speed benefit was accompanied by a substantial increase in NoGo errors, the critical measure for response inhibition. In contrast, no pronounced differences between 5-HTTLPR genotype groups were observed for homozygous carriers of the Val-allele of *BDNF*, neither for response latency nor for error rate. Thus, although Go RT itself cannot be regarded as a direct measure of inhibition, the shorter response latencies to Go stimuli at the expense of accuracy to NoGo events in homozygous l-allele carriers suggests a more impulsive response pattern relative to those carrying the s-allele of 5-HTTLPR. However, *BDNF* appears to have a moderating role, insofar as this response pattern was only evident in presence of the Met-allele. Conversely, the Val/Val genotype of *BDNF* might act as a protective factor that seems to compensate for erroneous (impulsive) responses linked to the l/l genotype of 5-HTTLPR.

Supporting these findings, similar interactions between 5-HTTLPR and *BDNF* Val66Met were observed for the Stop-Signal task. However, whereas interactions in the Go/NoGo task only point to general effects on task performance and thus were independent of the time of measurement, genotype-related differences in the Stop-Signal task were detected in the post-/follow-up testing sessions (T2/T3). Hence, given that no genotype-dependent performance differences were evident at pre-testing, such performance changes may partly be explained by training, or more general, by the repeated exposure to the task. Although all genotype groups showed a decrease in RT that was accompanied by an increase of commission errors at T2/T3, there were genotype-related differences in the pattern of speed and accuracy changes. Consistent with the Go/NoGo results, homozygous carriers of the l-allele of 5-HTTLPR exhibited shorter response latencies to Go stimuli than s-allele carriers if they carried the Met-allele form of *BDNF*. Again, this decrease in RT was accompanied by an increase in erroneous responses to Stop stimuli being reflected in the post-/follow-up testing sessions (T2/T3), while no such genotype-related differences were found between the 5-HTTLPR genotype groups carrying the Val/Val form of *BDNF*. Thus, the presence of Val/Val appears to protect against impulsive response patterns in l/l genotype carriers of 5-HTTLPR, replicating the Go/NoGo results.

In general, the present results on *BDNF* Val66Met fit with evidence on the functional role of *BDNF*. Specifically, it has been shown that the Met-allele is associated with impaired intracellular trafficking and packaging of the pro-form of the BDNF protein and a markedly reduced activity-dependent secretion of BDNF, while the Val/Val form leads to upregulated BDNF activity (Egan et al., 2003; Chen et al., 2004). Accordingly, Val/Val genotype carriers have been reported to show superior task performance than Met-allele carriers, notably on error- or accuracy-based measures such as in memory and learning tasks, which has been linked to Met-allele-related deficiencies in hippocampal early- and late-phase LTP (Egan et al., 2003; Bath and Lee, 2006; Dincheva et al., 2012). Similar effects have been observed in a variety of executive function tasks (Dincheva et al., 2012; see also Mon et al., 2013). These behavioral effects are backed up by relatively reduced gray matter volumes and aberrant BOLD responses in hippocampal and PFC areas in Met-allele carriers compared to those homozygous for the Val-allele (Hariri et al., 2003; Pezawas et al., 2004; Szeszko et al., 2005; Schofield et al., 2009).

However, as an important theoretical aspect of training, genotype-dependent performance changes should emerge at later measurement points, while no such changes would be expected at T1 (i.e., time effects). Indeed, the observed time × gene-gene interactions in the Stop-Signal task may be supported by the role of BDNF in mediating short- and long-term changes (i.e., plasticity) on neuronal and synaptic function, as a result of environmental or task-related demands (Poo, 2001; Binder and Scharfman, 2004; Cohen-Cory et al., 2010). Particularly in the Stop-Signal task, to maximize speed appears to be counterproductive as too fast responding inevitably goes along with a lower likelihood to inhibit the go-process in the face of the stop-signal leading to a higher rate of commission errors. Proceeding from this, the less pronounced decrease in RT and increase in commission errors as observed for 5-HTTLPR l-allele individuals carrying the *BDNF* Val/Val genotype can be seen as a more adaptive behavior, which is in line with the role of Val/Val in cognitive performance. In fact, the present results add to similar time or delay effects that were observed in recognition and episodic memory tasks demonstrating that Val/Val individuals have an advantage over Met-allele carriers such as in detecting previously encoded word stimuli and in maintaining these benefits across subsequent delays. This was interpreted in the context of rapid *BDNF* Val66Met-induced plasticity promoting stimulus acquisition/encoding and memory consolidation of task-relevant stimuli that in turn yielded a higher hit rate or a lower decline of retrieved target words in Val/Val carriers across later delays (see Goldberg et al., 2008; Montag et al., 2014).

In view of the role of 5-HTTLPR in these gene-gene interactions, several studies suggest that l/l genotype carriers performed less well than s-allele ones in a range of tasks that challenge the ability to focus on task-relevant information and to prevent competing or irrelevant information from gaining control over behavior. This includes 5-HTTLPR-related performance effects on target accuracy, response latency and measures of interference control, as, for example, derived from WM-, flanker- or inhibition tasks (Enge et al., 2011; Homberg and Lesch, 2011; Anderson et al., 2012; Enge et al., 2014b), which is supported by similar results in animals (Brigman et al., 2010; Jedema et al., 2010). There is also evidence suggesting lower hippocampal-dependent memory performance associated with the l/l-genotype of 5-HTTLPR (Roiser et al., 2007). Thus, on a single gene level, the time × 5-HTTLPR effects in the Stop-Signal task may provide some evidence for such previous findings. Notably, however, the results of both IC tasks consistently indicate that the more impulsive response pattern in l/l carriers (i.e., relatively increased false alarms compared to s-allele carriers) is primarily driven by the presence of the Met-allele of *BDNF*, but is substantially reduced for the BDNF secretion-enhancing Val/Val genotype. This protective role of Val/Val seems consistent with accumulated data that link this genotype with more accurate responses to task-relevant information relative to the Met-allele as reviewed above and further points to the additional value that can be gained from *BDNF* × 5-HT gene interactions.

Indeed, there is considerable evidence of BDNF and 5-HT system interactions to induce neurodevelopmental changes on long-term neuronal function (Homberg et al., 2014) and, regarding the present study, one may argue that such long-term effects could have contributed to the genetic interactions independent of time. However, the interplay of BDNF and 5-HT is not restricted to neurodevelopmental changes but is also evident during adulthood, such as by inducing rapid synaptic alterations in hippocampal regions, depending on current environmental demands (Mattson et al., 2004; Martinowich and Lu, 2008; Homberg et al., 2014). Thus, such rapidly induced synaptic changes could be reflected in the present time × gene-gene interactions leading to performance differences at T2/T3, as observed for RT and commission errors in the Stop-Signal task.

However, this genotype-related performance differences from pre- to post-testing did not directly occur as a result of the training intervention as there were no genotype-related differences between the adaptive training group and the active controls, neither in the Go/NoGo nor in the Stop-Signal task. From a methodological viewpoint, this suggests that no true changes in the trained IC function have occurred. However, as outlined above, there are genotype-dependent performance differences at the post-testing sessions (T2/T3) that might be interpreted as a result of training (or of the exposure to the tasks), given that no such differences could be observed at T1. Nonetheless, this also means that the IC training in consideration of important genetic moderators of brain plasticity did not lead to significant differences between the relevant experimental groups.

How might this be explained? One may speculate that being exposed to the tasks such as during the practice trials and the pre-testing session (conducted by all experimental groups) may have been sufficient to rapidly induce genetically-driven plasticity effects such as related to a fast, hippocampal-dependent acquisition and consolidation of task-specific demands, stimuli and response sets. This notion would be in line with evidence on the functional characteristics of BDNF and BDNF × 5-HT interactions being critically involved in rapid, activity-dependent alterations on synaptic properties in hippocampal regions, as a function of current environmental demands (Martinowich and Lu, 2008; Cohen-Cory et al., 2010; Bekinschtein et al., 2014; Homberg et al., 2014). Thus, one may argue that the lack of genotype-dependent differences between the three experimental groups reflect ceiling effects that could be due to the fact that a relatively low degree of exposure to the tasks might already be sufficient to establish genotype-dependent performance differences in all experimental groups, thereby reducing the chance of such differences between the experimental groups due to training.

Although the notion that the observed effects may reflect changes in hippocampal pathways should be considered as preliminary, it seems to be well supported by evidence on BDNF and 5-HT signaling to shape neuroplasticity in cortico-hippocampal regions and by the profound impact of BDNF-promoted plasticity in hippocampal-mediated learning and memory functions, as reviewed above. One may further argue that frontally based brain circuits that are more directly related to IC such as the inferior frontal cortex (Aron et al., 2004) are far less modifiable by short-term environmental influences such as induced by the present training. Conversely, hippocampal regions show extensive plasticity in response to internal and external (environmental) demands to promote adaptive behavior within a very short time range (Deng et al., 2010; Gu et al., 2013). Similarly, hippocampal-dependent practice or task-specific learning effects have been suggested as possible explanatory factors of cognitive training and transfer effects (Shipstead et al., 2010; Redick et al., 2013). Nonetheless, there is abundant evidence for the importance of the interplay between hippocampal and prefrontal “control” regions in establishing goal-directed behavior, demonstrating hippocampal regions to be coactivated and functionally coupled with prefrontal cortical areas during executive function tasks (Axmacher et al., 2007; Blumenfeld and Ranganath, 2007; Schofield et al., 2009; Whitney et al., 2009; Hyman et al., 2010; Brockmann et al., 2011; Depue, 2012).

## Limitations

Among the limitations of the present study, issues related to the training regime require attention. We had a 3-weeks training with nine training sessions, which is well in the typical range of trainings studies. Nevertheless, other studies have employed considerably more training sessions. However, recent evidence suggests that training transfer on non-trained abilities could not be detected irrespective of whether the training included 8 or 20 sessions (Chooi and Thompson, 2012; see also Melby-Lervåg and Hulme, 2013; Redick et al., 2013).

Moreover, several sample-related limitations also deserve mention. The first notion refers to our sample size of 122 individuals (of which 115 participated also at the T2/T3). This sample size is comparably large for studies on the effects of cognitive training, and it allowed us to detect three-way interaction effects of 5% explained variance at a significance level of 0.05 with a statistical power (1-β error probability) of 0.87. In the present study, we were especially interested in the three-way interaction Time × Group × 5-HTTLPR/BDNF as we expected individuals with the 5-HTTLPR s-allele or those homozygous for the BDNF Val-allele to benefit more from the IC training. However, the analyses particularly revealed 5-HTTLPR × BDNF interactions that—at least for the Stop-Signal task—were also time-dependent. Here, a more differentiated view on Time × Group × 5-HTTLPR × BDNF interactions would have been desirable. Our study had adequate power to detect and interpret three-way interactions, but not four-way interactions. Further, although the two IC tasks used here are commonly used as interchangeable markers of IC, there seem to exist subtle differences in the executive functions addressed as evidenced by overlapping, but partly different neural correlates (Swick et al., 2011). This could be one reason why significant time effects were only detected in the Stop-Signal task, but warrants further research. Moreover, sample size precluded more sophisticated data analysis approaches such as confirmatory factor analysis integrating task performance of the Go/Nogo and Stop-Signal task as the number of individuals was not sufficient to allow robust maximum likelihood estimation (see e.g., Boomsma and Hoogland, 2001). However, because in Enge et al. (2014a) performance changes were investigated for the Go/Nogo and Stop-Signal task separately, we aimed to investigate a possible moderating role of plasticity genes in a similar fashion to be as close as possible to this previous study. With regard to the observed gene-gene interactions, however, the sample size is a limiting factor. Although in both task, similar interactions were observed and thus effects were replicated across tasks, replication studies should strive for larger samples, in order to realize a more robust group size for single comparisons within genotype × genotype interactions.

Second, the imbalanced sex ratio (only about 15% male participants) may have impacted the results and thus may limit generalization of the effects observed here. Third, another sample-related issue may be effects arising from population stratification. However, our sample comprised students of Middle European descent that were born in Germany and reported German as their mother tongue. Therefore, a bias of the present results due to stratification effects is negligible. Fourth, in line with the standard protocol used in the training literature, we provided no individual feedback on task performance, neither during training sessions, nor at pre-, post- and follow-up test session. Therefore, the motivation to complete the tasks with high performance might have differed between subjects, which in turn might have led to rather small effects. While there were neither mood differences among the experimental groups nor genotype-related mood effects, a more specific measure of motivational differences would be desirable in future studies.

## Conclusion

In sum, the present results suggest that *BDNF* Val66Met and 5-HTTLPR modulate neuroplasticity in IC performance over time but independent of IC training, because there were no differences between the training groups. In line with ample evidence on BDNF and BDNF-5-HT system interactions to shape neuroplasticity especially in hippocampal regions, the findings may reflect genotype-dependent differences in the acquisition and memory consolidation of task-relevant information. This, in turn, may have led to a more adaptive responding in the tasks, particularly in those individuals carrying the Val/Val form of *BDNF* and the s-allele of 5-HTTLPR that have been previously associated with better cognitive performance.

## Author Contributions

SE planned and supervised the study and mainly wrote the manuscript. MF mainly carried out the statistical analyses and wrote parts of the results. KPL and AR did the genotyping and wrote parts of the genotyping section. AG participated in data collection and commented on drafts. MK helped to design the study and commented on drafts. AS planned the study, supervised the study and commented on drafts.

## Conflict of Interest Statement

The authors declare that the research was conducted in the absence of any commercial or financial relationships that could be construed as a potential conflict of interest.

## Footnotes

^1^www.neurobs.com

^2^Note that mean RT on Go-trials in both tasks was considered in addition to commission error rate as a more specific indicator of response inhibition in order to investigate the interplay between fast and accurate responding. Rapid responses to go-stimuli can lead to a lower likelihood to inhibit the no-go stimulus or the go-process in the face of the stop-signal leading to a higher rate of commission errors. Further note that accuracy for Go stimuli was nearly 100% and thus, commission error rate was virtually identical to the so-called d-prime measure.

^3^Note that no significant genotype-related effects occurred when stop-signal RT was used as behavioral indicator (all *p* > 0.10).
