# Supplementary material for: Brain-Derived Neurotrophic Factor (Val66Met) and Serotonin Transporter (5-HTTLPR) Polymorphisms Modulate Plasticity in Inhibitory Control Performance Over Time but Independent of Inhibitory Control Training
Source: Front Hum Neurosci. 2016 Jul 29;10:370. doi: 10.3389/fnhum.2016.00370 (PMC4966207; doi:10.3389/fnhum.2016.00370)
Supplement: Supplementary file 1 [file Image_1.pdf]

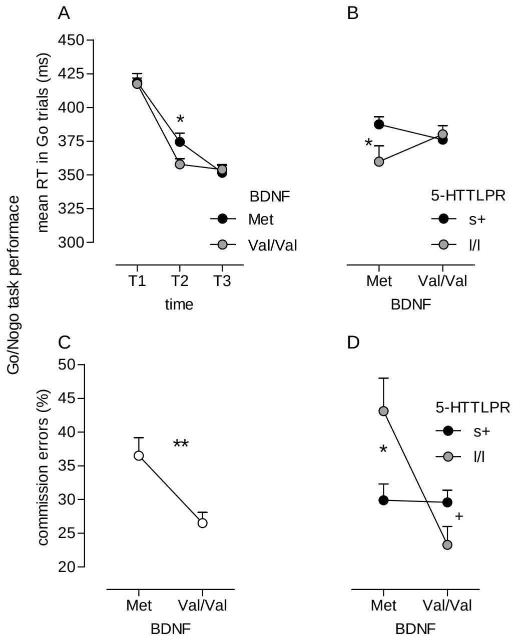

Figure 1. Go/NoGo task performance: Interaction of (A) Time x BDNF ($F_{2,180.}$ = 3.67, *p* = .035, η_p^2 = .04, ε = .84), and (B) 5-HTTLPR x BDNF ($F_{1,90}$ = 4.36, *p* = .040, η_p^2 = .05) on mean reaction time (RT) in Go trials as well as (C) main effect of BDNF ($F_{1,90}$ = 9.98, *p* = .002, η_p^2 = .10) and (D) interaction of 5-HTTLPR x BDNF ($F_{1,90}$ = 9.46, *p* = .003, η_p^2 = .10) on commission error rate (in %); N = 100; $^+p < .10$, $^*p < .05$, $^{**}p < .01$.

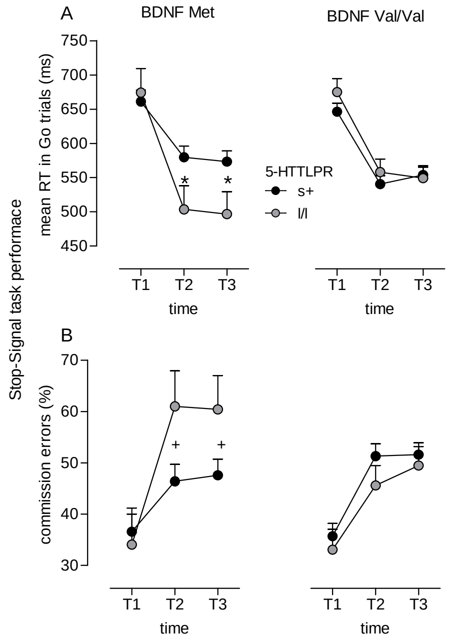

Figure 2. Stop Signal task performance: Interaction of (A) Time x 5-HTTLPR x BDNF ($F_{2,184}$ = 3.65, *p* = .036, η_p^2 = .04, ε = .84) on mean reaction time (RT) in Go trials; and of (B) Time x 5-HTTLPR x BDNF ($F_{2,184}$ = 4.82, *p* = .013, η_p^2 = .05, ε = .86) on commission error rate (in %); N = 104; $^+p < .10$, $^*p < .05$.
